# Comparison of endoscope-assisted and microscope-assisted type I tympanoplasty; a systematic review and meta-analysis

**DOI:** 10.1007/s00405-023-08305-1

**Published:** 2023-11-15

**Authors:** Kirolos Botros Elnahal, Mohamed Amir Hassan, Ahmed Mahmoud Maarouf

**Affiliations:** 1 Elmabarra Health Insurance Hospital, 16th Elthaora Street, Nile Corniche, Assiut, Egypt; 2 Faculty of Medicine, Ain Shams University, Cairo, Egypt

**Keywords:** Meta-analysis, Tympanoplasty, Myringoplasty, Endoscope, Microscope, Tympanic membrane perforation

## Abstract

**Objectives:**

To analyze and compare the available data about the outcomes of endoscopic and microscopic type I tympanoplasty.

**Data sources:**

PubMed, Cochrane library Ovid, Scopus, Google scholar, and ClinicalTrials.

**Methods:**

We conducted a meta-analysis in accordance with the Preferred Reporting Items for Systematic reviews and Meta-Analyses (PRISMA) guidelines. We included comparative studies describing type I tympanoplasty, and comparing surgical outcomes of the endoscope with the microscope in terms of efficacy and safety.

**Results:**

Our systematic search yielded 22 studies meeting the inclusion criteria and eligible for analysis. The pooled graft uptake rates and audiological results of endoscopic and microscopic tympanoplasty demonstrated non-significant differences. In contrast, endoscopic type I tympanoplasty outperforms microscopic tympanoplasty regarding a highly significant decrease not only in pooled mean operative time but also in the pooled complications rate.

**Conclusions:**

Based on our meta-analysis, the surgical outcomes of endoscope-assisted and microscope-assisted type I tympanoplasty in terms of postoperative hearing outcomes and the graft uptake rate were comparable. On the contrary, operative time and complications rate proved to be significantly reduced with endoscopy compared to microscopy. Hence, the endoscope is as efficient as the microscope in type I tympanoplasty but less invasive, fewer in complications and shorter in operative time.

## Key Points

• Can the endoscope be a good alternative to the microscope in type I tympanoplasty?
• Using the microscope in tympanoplasty has been the conventional procedure for repairing perforated tympanic membranes since the 1950s. However, ear surgeons have increasingly practised endoscope-assisted tympanoplasty since the late 1990s.
• In this study, surgical outcomes of both techniques in terms of postoperative audiological results and the graft uptake rate were comparable.
• In contrast, the endoscopic technique was superior to the microscopic one in terms of operative time and complications rate.
• According to our study, the endoscope-Assisted type I tympanoplasty proved to be as effective as the microscopic technique but safer and less invasive.

## Introduction

Tympanoplasty is a surgical procedure aiming at the eradication of infection, repair of the perforated tympanic membrane (TM), and hearing rehabilitation in patients with chronic otitis media (COM) [1]. Middle ear infections, trauma or iatrogenic injury are the principal causes of TM perforation. Up to 80% of TM perforations heal spontaneously [2]; as for the remaining, surgical repair is usually required [3].

With endoscope assistance, minimally invasive techniques of ear surgery have arisen and evolved since the 1990s [4]. Analogous to functional endoscopic sinus surgery, so too did the concept of functional endoscopic ear surgery (FEES). The philosophy of FEES fundamentally supports three essential principles: 1. using the external auditory canal (EAC) as the natural conduit to the tympanic cavity; 2. restoring normal ventilation routes of the middle ear and the mastoid; and 3. conserving as much normal anatomy as possible [5]. Consequently, endoscopic ear surgery has become widely accepted with anatomical and physiological concepts [6].

Despite the well-known merits of endoscopic techniques, some concerns about their efficiency and safety are still exist among some ear surgeons and hinder the transition from conventional microscopic tympanoplasty to endoscopic tympanoplasty for those surgeons [7].

Therefore, there is a need for a meta-analysis comparing the outcomes of both endoscopy and microscopy techniques of type I tympanoplasty in terms of efficacy and safety.

### Objectives

In this study, we aimed to make a comparison between endoscopic and microscopic type I tympanoplasty in relation to the duration of surgery, outcomes and complications through a meta-analysis.

## Materials and methods

We conducted a meta-analysis using the standard methodology outlined in the Cochrane Handbook [8] and reported the findings in accordance with the Preferred Reporting Items for Systematic reviews and Meta-Analyses (PRISMA) statement guidelines. [9] A PRISMA flow diagram was used to describe the flow of information through the various phases of the systematic review.

### Eligibility criteria

Inclusion criteria for our meta-analysis were as follows: (1) journal articles published in English; (2) articles concerned with TM perforation especially due to COM; (3) studies describing myringoplasty or type I tympanoplasty, and comparing surgical outcomes of the endoscope with the microscope; (4) either temporalis fascia or perichondrium as a source of the graft tissue; (5) graft placement method via underlay technique.

Exclusion criteria for our meta-analysis were as follows: (1) articles describing other types of otitis media (e.g. acute OM or OM with effusion) or other pathologies (e.g. cholesteatoma or middle ear tumours); (2) the aid of an endoscope holder; (3) cadaver studies; (4) animal studies; (5) irrelevant publications to our study.

### Outcome measures

The outcome measures, which we considered in terms of efficacy and safety, were average operative time (intraoperative outcome); average postoperative air-bone gaps (ABGs) improvement and graft uptake rate (primary efficacy outcomes); complications rate (secondary safety outcomes).

### Search strategy

We performed a systematic search for all available studies comparing surgical outcomes of the endoscope with the microscope in the databases of PubMed, Cochrane library Ovid, Scopus, Google scholar, and ClinicalTrials; dating from inception until 22 November 2019. We used the following keywords (in different combinations): microscopic, endoscopic, type I tympanoplasty, myringoplasty, chronic otitis media. Review articles and bibliographies of each identified study were searched for additional references that may contain further related studies.

### Study selection

Abstracts of articles identified using the above search strategy were reviewed; articles that appeared to fulfill the inclusion criteria were retrieved in full. We excluded duplicate records and irrelevant reports at this stage. When there was a doubt, a second reviewer assessed the article, and a consensus was reached.

### Data extraction

Data were independently extracted onto a previously edited Excel table by two reviewers and cross-checked; any discrepancies were resolved by consensus. For the meta-analysis, we retrieved the following information: author, year of publication, study design, number of patients, and outcomes regarding efficacy and safety.

### Statistical analysis

Data entry, processing and statistical analysis were carried out using Review Manager 5.3 (RevMan 2014) [10]. A meta-analysis was performed to calculate direct estimates of each treatment technique. Interventions for patients, who achieved favourable outcomes, were pooled to evaluate efficacy that was measured by standardized mean difference (SMD) with a 95% confidence interval (CI) for operative time and postoperative ABG improvement; and odds ratio (OR) with 95% CI for graft uptake rate. In addition, interventions for patients, who reached serious adverse events, were pooled to evaluate safety that was measured by OR with 95% CI for complications rate.

According to the heterogeneity of treatment effect across trials using the Chi^2^ test results and *I*^2^-statistics; a fixed-effect model (*P* ≥ 0.1) or random-effects model (*P* < 0.1) was used. In addition, we used a random-effects model for subgroup analysis.

### Assessment of risk of publication bias across studies

We assessed the publication bias across studies using the funnel plot method for each pooled analysis that included more than or equal to 10 studies [11].

## Results

### Study selection

**Fig. 1:**
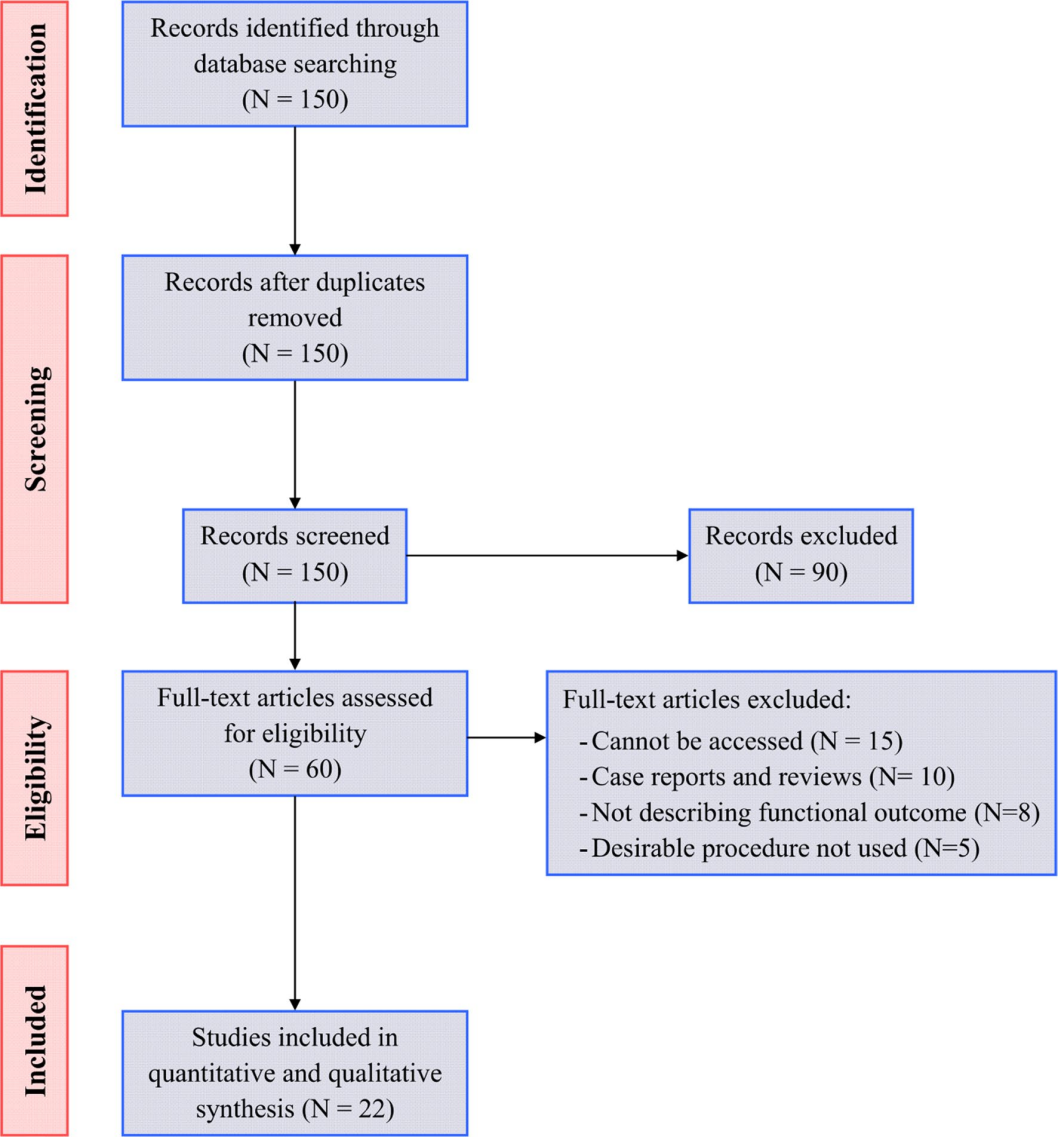
PRISMA flow diagram for study selection

Figure 1 represents the PRISMA flow diagram for the review process and study selection. We found 150 records by searching the database; of these, sixty records were left after removing the duplicates and after the exclusion of ninety records based on the title and the abstract review. We searched 60 articles for eligibility by full-text review; 15 articles cannot be accessed or obtain full-text; 10 studies were reviews and case reports; eight studies did not describe the functional outcome; the desired procedure was not used in five studies leaving 22 studies [12–33] that met all inclusion criteria.

### Study characteristics

Table 1 shows the demographic characteristics and clinical data of all 22 studies [12–33]. Regarding their design, these 22 studies included ten randomized controlled trials, six prospective comparative studies, five retrospective comparative studies and one retrospective cohort study. The enrolled studies were published between 2008 and 2019. The total number of interventions was 1578 interventions; with 766 interventions in the endoscopic group, and 812 in the microscopic group.

**Table 1:** Summary of interventions and study characteristics

Author, year	Study design	Number of interventions		Comparative parameters			
		ET	MT	Operative time	ABG improvement	Graft uptake	Complications rate
Harugop et al., 2008 [12]	RCT	50	50	√		√	√
Lade et al., 2014 [13]	RCT	30	30		√	√	
Kumar et al., 2015 [14]	Prospective comparative	30	30	√	√	√	√
Nassif et al., 2015 [15]	Retrospective cohort	22	23	√	√	√	
Patel et al., 2015 [16]	RCT	22	22	√		√	√
Ahmed et al., 2016 [17]	Prospective comparative	50	50	√	√	√	
Gadag et al., 2016 [18]	RCT	30	30	√		√	
Gaur et al., 2016 [19]	Prospective comparative	30	30	√		√	√
Huang et al., 2016 [20]	Retrospective comparative	50	50	√	√	√	√
Kumar et al., 2016 [21]	RCT	30	30	√	√	√	√
Lakpathi et al., 2016 [22]	Prospective comparative	30	30	√		√	√
Sanji et al., 2016 [23]	Retrospective comparative	16	28	√		√	
Shoeb et al., 2016 [24]	Prospective comparative	30	30		√	√	
Choi et al., 2017 [25]	Retrospective comparative	25	48	√	√	√	
Jyothi et al., 2017 [26]	RCT	60	60	√	√	√	√
Kuo and Wu, 2017 [27]	Retrospective comparative	62	44	√	√	√	√
Sinha et al., 2017 [28]	RCT	22	22		√	√	
Khaliq et al., 2018 [29]	RCT	30	30	√	√	√	
Maran et al., 2018 [30]	RCT	30	30	√	√	√	
Saggu et al., 2018 [31]	Prospective comparative	30	30	√		√	
Ohki et al., 2019 [32]	Retrospective comparative	47	75	√	√	√	
Sundararajan et al., 2019 [33]	RCT	40	40	√	√	√	√

*ET* endoscopic tympanoplasty; *MT* microscopic tympanoplasty; *ABG* air–bone gap; *RCT* randomized controlled trial

## Effects of interventions

### Operative time

**Fig. 2:**
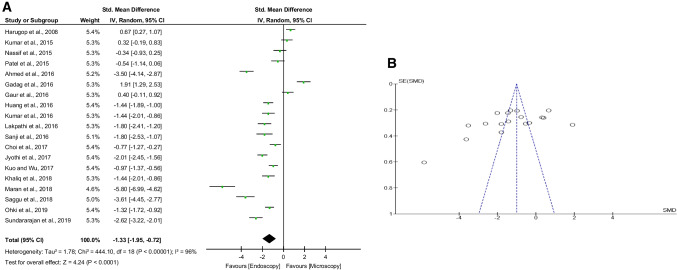
**A** Forest plot comparing the operative time of endoscopic tympanoplasty and microscopic tympanoplasty. **B** Funnel plot of the operative time. *CI* confidence interval; *IV* inverse variance; *SE* standard error; *SMD* standardized mean difference

We found nineteen studies that reported operative time with a total number of interventions (*n* = 1414). The average operative time was (77.7 ± 24.5) min for the ET group and (91.7 ± 18.8) min for the MT group. In the pooled analysis (Fig. 2A), endoscopic tympanoplasty (ET) showed a highly significant decrease in mean operative time compared to microscopic tympanoplasty (MT) (SMD: −1.33; 95% CI −1.95 to −0.72; *p* < 0.0001). However, highly significant heterogeneity (*I*^2^ = 96%, *p* < 0.00001) and publication bias were found (Fig. 2B).

### ABG improvement

**Fig. 3:**
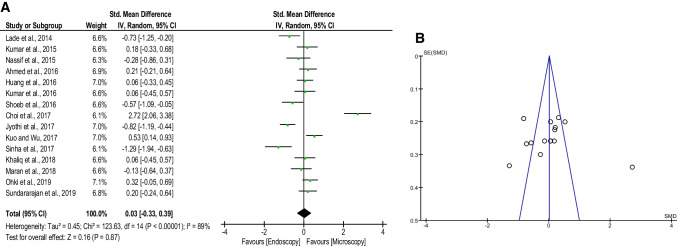
**A** Forest plot comparing the air-bone gaps improvement of both techniques. **B** Funnel plot of the air-bone gaps improvement. *CI* confidence interval; *IV* inverse variance; *SE* standard error; *SMD* standardized mean difference

Fifteen studies exhibited ABG improvement with a total number of interventions (*n* = 1135). Based on the pooled analysis (Fig. 3A), ET showed a non-significant difference in mean ABGs improvement compared to MT (SMD: 0.03; 95% CI −0.33 to 0.39; *p* = 0.87). However, highly significant heterogeneity (*I*^2^ = 89%, *p* < 0.00001) and publication bias were found (Fig. 3B).

### Graft uptake rate

**Fig. 4:**
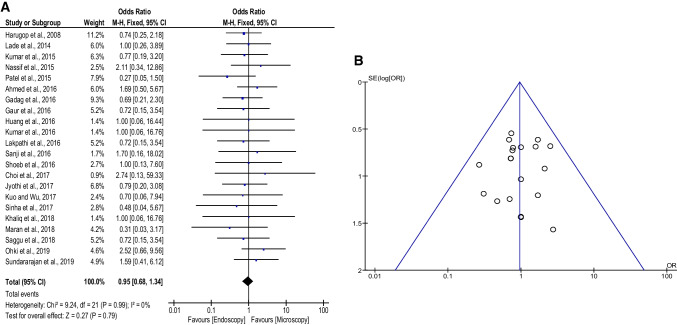
**A** Forest plot comparing the graft uptake rate of endoscopic tympanoplasty and microscopic tympanoplasty. **B** Funnel plot of the graft uptake rate. *CI* confidence interval; *M*–*H* Mantel–Haenszel; *OR* odds ratio; *SE* standard error

All 22 studies included data about graft uptake rate with a total number of interventions (*n* = 1559). Accrued results (Fig. 4A) showed that ET was as effective as MT (89.8% vs. 90.2%; OR: 0.95; 95% CI 0.68–1.34; *p* = 0.79). In addition, no heterogeneity (*I*^2^ = 0%, *p* = 0.99) or publication bias was found (Fig. 4B).

### Postoperative complications

Various postoperative complications were retrieved from enrolled studies. We analyzed the incidence of postoperative complications, with a particular focus on the infection (wet ear), wound gap, visible scar, nausea and vomiting. Incidence of postoperative complications was reported in 10 studies.

Data regarding the infection (wet ear) were available from six studies with a total of 486 interventions. Three studies reported data on the wound gap with a total of 210 interventions. Based on the pooled analysis, ET showed a non-significant difference compared to MT in terms of the rates of infection (6.3% vs 7.3%; OR: 0.94; 95% CI 0.46–1.94; *p* = 0.87) and wound gap (0.9% vs 6.3%; OR: 0.25; 95% CI 0.05–1.30; *p* = 0.10). Also, no heterogeneity was found.

Data regarding visible scar, we found three studies with a total of 220 interventions. Two studies reported data on postoperative nausea and vomiting, with a total of 160 interventions for each. In the pooled analysis, ET showed a highly significant decrease compared to MT in terms of the rates of visible scar (0% vs 72.7%; OR: 0.01; 95% CI 0.00–0.03; *p* < 0.00001), nausea (36.3% vs 67.5%; OR: 0.27; 95% CI 0.14–0.53; *p* = 0.0001), and vomiting (12.5% vs 43.8%; OR: 0.18; 95% CI 0.08–0.41; *p* < 0.0001). In addition, no heterogeneity was found.

**Fig. 5:**
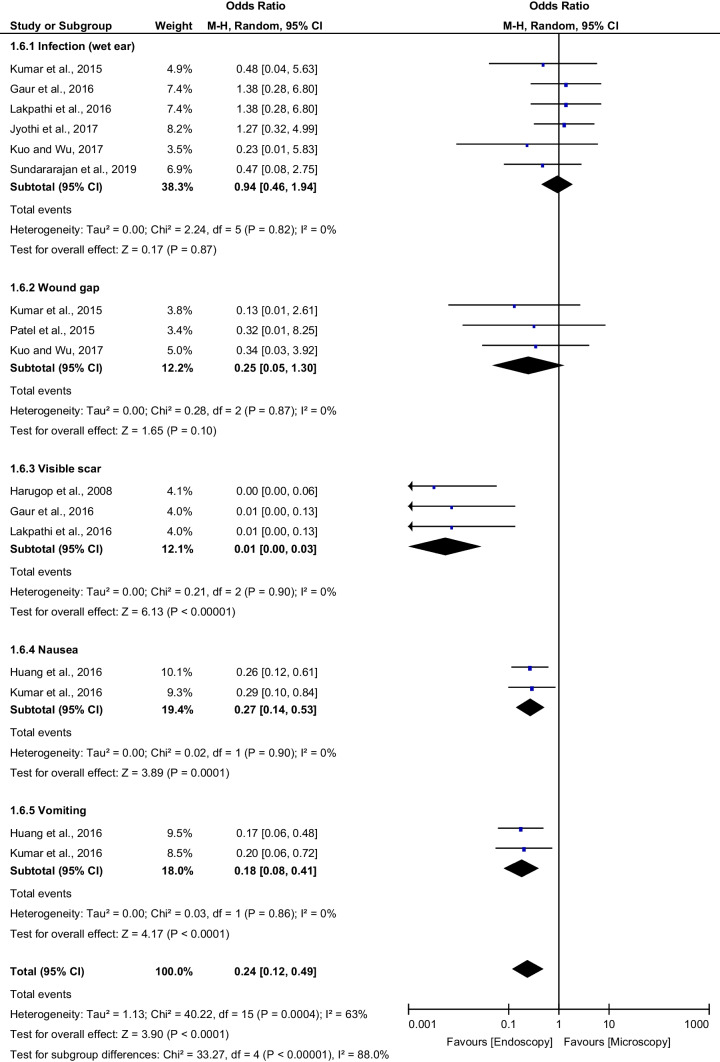
Forest plot comparing the postoperative complications rate of endoscopic tympanoplasty and microscopic tympanoplasty with regard to infection (wet ear), wound gap, visible scar, nausea and vomiting. *CI* confidence interval; *M*–*H* Mantel–Haenszel

The overall effect of pooling the previous five subgroups showed that the rate of complications is significantly lower in ET than in MT (8.8% vs 32%; OR: 0.24; 95% CI 0.12–0.49; *p* < 0.0001) (Fig. 5).

## Discussion

In this study, we compared the outcomes of endoscopic with microscopic type I tympanoplasty through a systematic review meta-analysis. Hearing improvement and graft uptake rate of ET were comparable to those of MT. On the other hand, ET was superior to MT in terms of operative time and complications.

For decades, Microscope-assisted surgery was the main modality for ear surgery, allowing two-handed manipulation, binocular vision and an excellent stereoscopic surgical view. However, the vision of a microscope is along a straight line and may be limited in hidden areas and the deep recesses of the middle ear, so the surgeon has to use the post-auricular approach instead of the transcanal approach to obtain a wider surgical view.

One of the primary advantages of the endoscope is the panoramic and wide-angle surgical view with magnification. The endoscope can be approximated to the surgical field, bypassing the narrowing parts of the EAC, and the angled endoscope can be rotated to obtain all-round vision without the requirement of these invasive maneuvers, thereby reducing morbidity and operative time. In contrast, the microscope has a straight-line view, which can be limited when encountering variations of the EAC, such as a tortuous, stenotic ear canal and bony overhangs. Therefore, surgeons may need to drill out or curette bony overhangs during canaloplasty and canal wall curettage for complete visualization and assessment of the TM and the status of ossicles.

Endoscope-assisted surgery provides a wide field of view with magnified images, uses a smaller surgical incision, and preserves more tissue. In addition, endoscopes with different angles enable ‘‘around the corner’’ visualization of hidden areas and middle ear recesses. However, the endoscope lacks binocular vision (i.e. lost depth perception) and requires training besides being a one-handed technique, and therefore it is difficult with limited value in case of excessive bleeding in which the blood soils the tip of the endoscope obscuring the surgical field. Moreover, neck strain and backache related to direct vision through the endoscope and arm fatigue due to the weight of the scope and its camera may be demerits that can be overcome by developing a stand for the endoscope.

### Comparable effects concerning graft uptake rate and hearing improvement results

With regard to the graft uptake, no significant difference was found between ET and MT. Similar results were also reported in previous meta-analytic studies of Tseng et al. [34], Lee et al. [35] and Pap et al. [36]. In this meta-analysis, we selected included studies that used similar operative techniques such as grafting material (temporalis fascia or perichondrium) and the graft placement method by underlay technique to obtain more accurate results about graft uptake rate.

Audiological results resembled graft uptake outcomes. Not unexpectedly, ABGs improvement demonstrated no significant difference between ET and MT, despite discrepancies in hearing evaluations. Remarkable TM closure rates between ET and MT may explain comparable audiological outcomes. However, potential publication bias with highly significant heterogeneity may have negatively impacted the integrity of this analysis. Tseng et al. [34], Lee et al. [35] and Pap et al. [36] reported similar analytic results.

### Advantages of ET over MT

In agreement with Lee et al. [35] and Pap et al. [36], another significant advantage regarding ET is that the operative time for ET was significantly shorter than for MT. The surgeon's experience and the learning curve generally have an impact on the operative time. However, MT consumes more time due to frequent manipulation of the patient’s head or repeated microscope adjustment for a better view, using the post-auricular approach, or performing canaloplasty and curettage. According to Hsu et al. the relatively short time required for surgery and under anaesthesia results in significantly fewer medical resources expended on ET and decreased complications from prolonged exposure to anaesthesia [37]. In our meta-analysis, the analysis for operative time data suffers from significant heterogeneity and publication bias.

Characteristically, ET is advantageous concerning safety, minimal invasiveness and the rate of complications. Because of a wide field of view with magnification, ear surgeons have obtained minimally invasive endoscope-assisted tympanoplasty accompanied by minimal complications. In our meta-analysis, we focused particularly on the following complications: the infection (wet ear), wound gap, visible scar, nausea and vomiting. No significant difference was found between both techniques with regard to infection and wound gap, but there was a highly significant decrease in the rates of visible scar, nausea and vomiting as well as the overall complications rate in favour of ET. Postoperatively, the wet ear results from a severe middle ear infection [37] and the wound gap following suture removal from early loose stitches [27] rather than the procedure itself. In the present study, a meta-analysis of cosmetic results through the presence or absence of a visible scar revealed that the endoscope was definitely preferred over the microscope. For ET, the transcanal approach to the middle ear and smaller incision with minimum tissue dissection for harvesting a graft lead to early wound healing and less scarring on the graft donor site [16, 18, 26]. Besides, avoiding the post-auricular route reduces the incidence of auricular displacement and asymmetry of the pinna yielding better cosmetic outcomes [12, 14, 19, 22]. Similar to meta-analytic results of visible scar, the rates of nausea and vomiting were significantly lower after ET than after MT. Nausea and vomiting are unpleasant events and are associated with patient discomfort and dissatisfaction during postoperative recovery [38]. These two adverse events require the administration of various treatment modalities and consequently can expand recovery room time, increase nursing care requests and the duration of hospital stay, and can further increase total healthcare costs [39].

In concordance with our results regarding complications, Lee et al. [35] reported that wound problems of ET were significantly lower than those of MT, but there was no significant difference between ET and MT regarding wet ear.

### Strengths and limitations

Our meta-analysis possesses several strengths. Our findings were comparable to those presented in the previously published meta-analyses [34–36]. Unlike the preceding meta-analytic publications about the same topic, the present study included more randomized controlled trials and other comparative studies that the search had yielded. Aiming at a better assessment of efficacy and safety, we also added more parameters for comparison. As much as we could, we held some variable risk factors constant, such as the source of the graft tissue and the graft placement method, to reduce clinical heterogeneity.

Admittedly, our meta-analysis has a few limitations. There was a noticeable variance in the other risk factors influencing surgical outcomes (e.g. the age of patients, and the size or location of TM perforation). This variance resulted in raising concerns about clinical heterogeneity. Furthermore, publication bias with highly significant heterogeneity could limit the integrity of our analytic results regarding operative time and ABGs improvement. Nevertheless, this study provided results that may be beneficial to decision-making and outcome prediction in patients receiving ET.

## Conclusion

Based on our meta-analysis, the surgical outcomes of endoscope-assisted and microscope-assisted type I tympanoplasty in terms of postoperative hearing results and the graft uptake rate were comparable. Operative time and complications rate, on the other hand, proved to be significantly reduced with endoscopy compared to microscopy. Hence, the endoscope is as efficient as the microscope in type I tympanoplasty but less invasive, fewer in complications and shorter in operative time.

Our results may be beneficial to decision-making and outcome prediction in patients receiving ET.

The current meta-analysis justifies the introduction of the endoscope to type I tympanoplasty and implies that the endoscope can be a better alternative to the conventional microscope technique. However, the potential effect of the location of TM perforation and the learning curve in surgical practice, besides other influencing factors, such as healthcare costs, intraoperative bleeding, postoperative hospital stay and the inner ear thermal damage, should be further investigated.

## Data Availability

Available.
